# Traditional Uses, Nutritional and Pharmacological Potentials of *Clerodendrum volubile*

**DOI:** 10.3390/plants10091893

**Published:** 2021-09-13

**Authors:** Kunle Okaiyeto, Ayodeji Osmund Falade, Oluwafemi Omoniyi Oguntibeju

**Affiliations:** 1 Phytomedicine and Phytochemistry Group, Department of Biomedical Sciences, Faculty of Health and Wellness Sciences, Cape Peninsula University of Technology, Bellville 7535, South Africa; okaiyetok@cput.ac.za; 2 Department of Biochemistry, Faculty of Basic Medical Sciences, University of Medical Sciences, Ondo 351101, Ondo State, Nigeria; ayodeji.falade@yahoo.com

**Keywords:** *Clerodendrum volubile*, magic leaf, leafy vegetable, pharmacological potentials, nutritional properties

## Abstract

*Clerodendrum volubile* is an underutilized leafy vegetable consumed in some parts of Nigeria. The interest in *C. volubile* has continued to increase due to its multipurpose values, including traditional uses, nutritional properties, and some therapeutic potentials; however, the pharmacological prospects of the plant are yet to be fully explored. Therefore, in the present review, different databases such as PubMed, Scopus, Web of Science, Google Scholar, etc. were explored to retrieve publications used to write this review. The pharmacological potentials of *C. volubile,* such as anticancer, antioxidant, antiviral, antimicrobial, anti-inflammatory, hepatoprotective, antidiabetic, and anti-hypertensive properties, were highlighted. The toxicological potential of the plant is also discussed. Proposed mechanisms that underline its biological activities include modulation of redox homeostasis, leading to decreased oxidative stress; down-regulation of matrix metalloproteinase-9 (MMP-9) expression; inhibition of key enzymes implicated in diabetes mellitus, hypertension, and neurological diseases; and inhibition of oxidative burst and inflammatory cytokines. Furthermore, the prospect of endophytes from *C. volubile* as a bioresource to produce novel therapeutic agents, as well as the development of nanotherapeutics from the plant extracts and its phytoconstituents, are discussed. In conclusion, *C. volubile* possesses an enormous number of possible pharmacological properties and therapeutic potentials waiting to be explored.

## 1. Introduction

**Figure 1:**
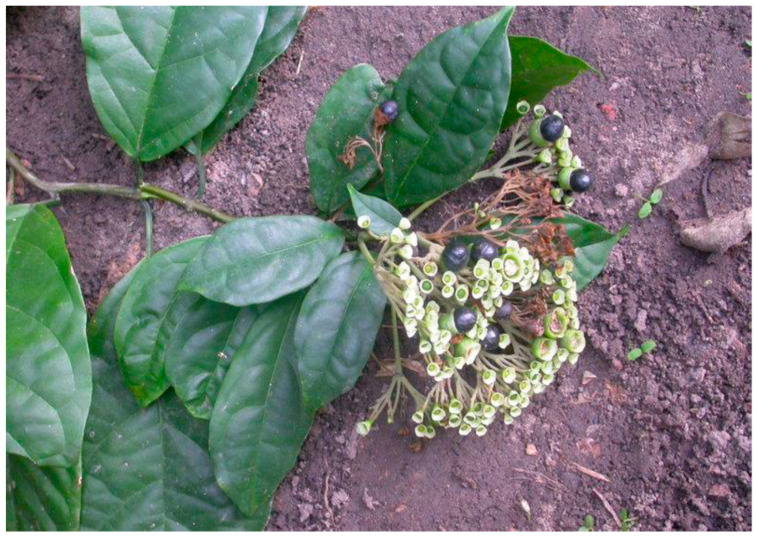
*Clerodendrum volubile* (Source: [16]).

Despite a long history of use of medicinal plants in developing countries around the world, scientific research into their beneficial health effects only gained popularity over the last few decades [1]. The field of complementary and alternative medicines support the use of medicinal plants and nutraceuticals as therapeutic/chemopreventive agents and in combating resistance while ameliorating toxic side effects of several chemotherapeutic agents. Also, there is scientific evidence of their efficacy in preclinical and clinical models [2]. Consequently, the significance of medicinal plants cannot be underrated in the management and treatment of various life-threatening diseases [3]. Medicinal plants contain various bioactive compounds that either act individually, additively, or synergistically to improve health [4,5,6,7,8,9]. Several factors encourage the inclusion of herbal regimens in healthcare management in both developed and developing countries, including affordability, availability, and accessibility [10,11]. Besides, the rural dwellers are less privileged and lack access to balanced diets, which has posed a high risk to their health and well-being, thus resulting in high malnutrition among children in the rural areas [4]. On the contrary, malnutrition problems can be unraveled by encouraging an increase in the consumption of healthy foods that are rich in carbohydrates, proteins, energy, vitamins, and minerals. The lack of nutritional information and inadequate development of nutritionally improved products from local raw materials have a direct bearing on nutrition. Moreover, researchers have focused more on the exploration of seeds for therapeutic purposes, while neglecting leafy vegetables [12]. Interestingly, leafy vegetables are cheap, easy to cook, and they have been acknowledged as one of the major sources of antioxidants, vitamins, and minerals needed for the maintenance of human cells and tissues, as well as normal body functioning [10,11]. The lack of availability of healthy foods rich in vitamins and minerals is responsible for a worse development of cognitive abilities during growth in the youth of said countries [13]. A good example of the leafy vegetable is *C. volubile*, which has gained prominence among the medicinal plants with remarkable therapeutic uses, perhaps due to its multipurpose values in folk medicine and various nutritional attributes. *C. volubile* (Lamiaceae) is a widely distributed vegetable in the warm temperate and tropical regions of the world [12]. The plant is popularly known as “marugbo” or “eweta” amongst the Ikale, Ilaje, and Apoi people in the southern-senatorial district of Ondo State, southwest Nigeria. It is, however, referred to as “obnettette” in the south-southern part of Nigeria [14]. The leaf of *C. volubile* (Figure 1) is commonly consumed as a vegetable, mostly blended with other vegetables as a spice with a sweet aroma and taste [14]. In Nigeria and other West African countries, green leafy vegetables undergo a cooking process rather than being eaten raw. Cooking is usually carried out to increase the palatability and to improve the edibility of some food [15].

## 2. Methodology

The information used to write this review was obtained from the publications obtained from different databases, such as PubMed, Scopus, Web of Science, Google Scholar, etc. The scientific name “*Clerodendrum volubile*” was used to recover all relevant information, such as the traditional uses, nutritional compositions, phytochemical compounds, and pharmacological potentials (anticancer, antioxidant, antiviral, antimicrobial, anti-inflammatory, hepatoprotective, antidiabetic and anti-hypertensive) of the plant discussed in this review.

## 3. Traditional Uses of *C. volubile*

*C. volubile* is a promising source of minerals and vitamins, which can be used to fight malnutrition if correctly exploited [17]. The report of Adefegha and Oboh [18] has highlighted the presence of zinc and iron in the plant, which are crucial cofactors in various enzymatic reactions in the body. The minerals are significant in fresh skin maintenance [19]. In southwestern Nigeria, the plant is usually used to stimulate appetite and revitalize women after they have given birth. It is usually used in the management of arthritis, swellings, rheumatism, gout, dropsy, and oedema, while also possessing anti-abortifacient and sedative properties [20]. The plant had been used in the treatment of inflammation and pain by traditional medical practitioners, but with no scientific evidence to support this.

## 4. Nutritional Compositions of *C. volubile*

Proximate and nutrient analyses have been reported to play important roles in assessing the nutritional significance of edible plants and vegetables [21]. Notable among the vegetables with remarkable nutritional importance is *C. volubile*. The proximate analyses of *C. volubile,* as reported by Erukainure et al. [20], revealed a high percentage of crude protein and ash content (12.14%), and nitrogen-free extract (NFE) (11.2%), while the highest percentage was observed in dry matter (DM) content (93.3%). Likewise, mineral analysis of the vegetable revealed high contents of sulfur, chlorine, manganese, iodine, and zinc, with sulfur content being the highest (131.45 mg/kg). The vegetable is characterized by very high amounts of vitamin A, vitamin C, and vitamin B12, as well as moderately high amounts of vitamin B3 and vitamin B6. Similarly, proximate analyses of *C. volubile* assessed by Ogunwa et al. [19] revealed the presence of a high percentage of fiber (11.26%), protein (13.88%), carbohydrate (44.69%), and ash (11.67%) contents. The analyses of the mineral contents were Na (22.86 ± 1.38 mg/100 g), K (27.69 ± 3.59 mg/100 g), Ca (30.91 ± 1.14 mg/100 g), Mg (27.11 ± 0.85 mg/100 g), Zn (24.27 ± 5.29 mg/100 g), Fe (6.22 ± 0.67 mg/100 g), Cu (0.04 ± 0.01 mg/100 g, Mn (6. 25 ± 0.59 mg/100 g), and P (27.61 ± 0.71 mg/100 g). Furthermore, the amino acid analysis showed the presence of Leu 7.60, Glu 9.88, Asp 8.14, Val 3.95, Arg 4.68, Gly 3.25, Lys 3.82, Ser 2.35, Ala 3.81, Ile 2.97, Thr 2.98, and Phe 3.78 g/100 g protein. The findings from the proximate analyses showed that *C. volubile* could serve as a good nutritional candidate in reducing malnutrition [20]. Deficiency of essential nutritional elements such as vitamins, minerals, and others can deplete the endogenous antioxidants and negatively affect synthesis of endogenous antioxidant enzymes and their activities, which could consequently induce oxidative stress in the body [22]. Attention has also been geared towards research on the proximate analyses of leafy vegetables because of their richness in minerals and vitamins that could be partially responsible for the pharmacological uses [10,11].

## 5. Bioactive Compounds Responsible for the Biological Activities

Phytochemical profiles of *C. volubile* leaf extracts have shown that it contains several secondary metabolites such as saponins, alkaloids, anthraquinone, flavonoids, phenolics, and cardiac glycoside [23]. This was corroborated by Adefegha and Oboh [18], who reported the presence of flavonoids, alkaloids, tannins, phenols, and saponin in *C. volubile*. It has, therefore, been suggested that the bioactive compounds could be responsible for the various pharmacological activities observed in *C. volubile*. Some of the identified compounds include chlorogenic acid, caffeic acid, rutin, isoquercitrin, quercitrin, quercetin, and kaempferol, which could be responsible for the plant’s numerous biological activities [18]. Similarly, a study conducted by Ogunwa et al. [24] revealed the presence of saponins, flavonoids, and phenols, amongst other compounds. Other researchers also identified the presence of 5,7-dihydroxy-4′-methoxyisoflavone (biochanin) and kaempferol 5,7,4′-trimethyl ether [25]; protocatechuic acid [26]; pectolinarigenin [25]; and chlorogenic acid, caffeic acid, kaempferol, quercetin, quercitrin, isoquercitrin, and ellagic acid [18,25] in the plant.

Moreover, several studies have confirmed the biological activities of these phytochemicals in *C. volubile* [27,28]. Nevertheless, much attention has been given to the phenolic-rich extracts, possibly due to their significant antioxidant, anti-inflammatory, and anti-diabetic properties, as well as the cardioprotective property through inhibition of the angiotensin I-converting enzyme (ACE) [29,30]. The hydroxyl groups in the phenolics are accountable for their strong antioxidant activity [31,32]. These hydroxyl groups have the capacity to donate an electron to unstable free radicals. Besides, the delocalization of π-electrons in the benzene ring results from an interaction between the functional moiety and the benzene ring [33]. The interaction of phenolics with free radicals inhibits their consequential destructive effects, thus mitigating against oxidative damage in cells. In addition, phenolics can also chelate metal ions (such as Fe, Cu, Cr, and Co) that are usually involved in redox metabolism, thereby inhibiting the formation of free radicals through Fenton reaction, as well as lipid peroxidation and DNA fragmentation [34,35].

Oxidative stress is habitually triggered by reactive oxygen species (ROS) and reactive nitrogen species (RNS), which play significant roles in the pathophysiology of chronic and life-threatening diseases [36]. Normally, the function of antioxidants is to neutralize free radicals by donating electrons and helping to prevent cell and tissue damage [37]. The side effects of prolonged usage of synthetic antioxidants have encouraged the search for natural antioxidants. Antioxidant-rich foods, most especially their phenolic constituents, play a vital protective role against oxidative damage caused by free radicals [38]. Foods rich in natural polyphenol have shown several health benefits, such as the prevention of hyperlipidemia and oxidative-stress-related diseases [39]. Hence, research on antioxidant-rich foods, plants, and vegetables has been on the upward trajectory globally to find a permanent solution to many chronic diseases [40]. Most of the health-promoting and disease-curing/preventing potential of plants and their products are associated with their phytoconstituents [41]. The leaves are rich in phenolic content, which has been correlated with their reported biological activities [42].

## 6. Pharmacological Potentials of *C. volubile*

### 6.1. Anticancer Activity

Cancer is a global public health disease with a huge negative impact on the human population [43]. The disease is usually caused by uncontrollable cell divisions in the body, resulting in tumors of malignant cells with a high metastatic prospect [44]. The search for a novel and effective treatment option has been on the increase because of the adverse effects of synthetic therapeutic anticancer drugs [1]. Interestingly, plants produce secondary metabolites for protection during their growth and these compounds have been reported to have the ability to inhibit cancerous cells growth [45]. Thus, the field of alternative and complementary medicines has opened new ways to develop novel plant-based anticancer agents to combat the resistance of cancerous cells to the current chemotherapeutic agents. Consequently, research interest is being directed towards natural products because of their safety, as compared to the current chemotherapy [1,2,43].

The antiproliferative potential of *C. volubile* has been well studied and documented by some researchers in the literature. For example, Erukainure et al. [27] reported the antiproliferative activity of fatty acid from *C. volubile* leaves against MCF-7 human breast cancer cell lines. In the study, it was observed that the fatty acids considerably inhibited cell growth, arrested G0/G1 phase by down-regulating the gene (MMP-9) expression and mitigated against oxidative stress in MCF-7 cell lines. In another study by Erukainure et al. [46], the in vitro antiproliferative effect of the dichloromethane leaves extract of *C. volubile* was investigated against human embryonic kidney (HEK293) cells. The results revealed that the extract exhibited cytotoxic effects against HEK293 cells with a concurrent increase in proinflammatory, reduction of antioxidative biomarkers, and ATP depletion, leading to cell apoptosis. Likewise, fatty acids from *C. volubile* leaves exhibited antiproliferative activity against human glioblastoma multiforme (U87MG) cell lines [47]. Afolabi and co-workers [1] also reported the antiproliferative effect of methanol extract of *C. volubile* leaves against prostate cancer (PCa) cells. They observed that the extract was able to suppress clonogenic potential of PCa cells in a colony and 3-(4,5-dimethylthiazol-2-yl)-2,5-diphenyl-2H-tetrazolium bromide (MTT) assays. An increase in the levels of cyclin-dependent kinase inhibitor p21 signified the modulation of cell cycle machinery [1]. In addition, a concentration-dependent cleavage of PARP and Caspase 3 was observed through the Western blot analysis of the extract-treated cells. To buttress this fact, the authors performed flow cytometric analysis and the data generated from flow cytometric analysis confirmed that apoptosis was the main mechanism of the extract-induced cell death.

### 6.2. Antioxidant Activity

Oxidative stress plays a crucial role in vivo tissue damage; it is important for the pathogenesis of several life-threatening diseases, as well as in the ageing of living organisms, with documented scientific evidence to support this [48,49]. Endogenous antioxidant enzymes play a significant role in protecting the biomolecules of the living organism [50]. Excessive production of ROS leads to a redox imbalance between free radicals and endogenous antioxidants, thereby causing oxidative stress, leading to the destruction of proteins and the structural integrity of lipid membranes of cells, as well as DNA of organisms [51]. Hence, the need for exogenous antioxidants is highly imperative. Over the years, synthetic antioxidant drugs have been effectively employed for the treatment of diseases mediated by free radical species [52]. However, adverse effects associated with the usage of synthetic antioxidant drugs cannot be neglected; therefore, huge consideration has been given to natural sources of antioxidants, specifically those of plant origin. Among the bioactive compounds in plants, phenolic compounds have been given special attention because of their strong antioxidant effect [53]. As a result, several researchers have shown interest in the investigation of underutilized indigenous leafy vegetables as a source of micronutrients and antioxidants for fighting malnutrition in sub-Saharan Africa [20]. A study by Erukainure et al. [54] highlighted that the effectiveness of endogenous antioxidant defense systems is maintained by an adequate dietary intake of vitamins and trace minerals. A plethora of evidence of the antioxidant properties of *C. volubile* has been reported in the literature by Erukainure and co-workers [27,54,55]. The plant is characterized by rich micronutrients and high flavonoid contents that account for its strong antioxidant effects in a dose-dependent manner [20].

In addition, an iridoid monoterpene, ajugoside, was isolated from the *n*-butanol fraction of *C. volubile* by Erukainure and colleagues [56]. Ajugoside exhibited a strong antioxidant protective role through its ability to scavenge different radicals (DPPH and ^•^OH) in male Wistar rats’ tissues. The antioxidant property of the *C. volubile*-derived compound, ajugoside, was also evident in the observed increase in endogenous enzymes accompanied by a decrease in the by-product of lipid peroxidation [56]. Considering the IC_50_ values, free soluble phenolic extract (IC_50_ = 89.18 µg/mL) from *C. volubile* exhibited higher DPPH radical scavenging ability than the bound phenolic extracts (IC_50_ = 133.40 µg/mL). In the same vein, the free soluble phenolic extracts had higher OH radical scavenging activity (IC_50_ = 924.90 µg/mL) than the bound phenolic extracts (IC_50_ = 1224.0 µg/mL). The metal ion reducing power of the extract was assessed and the results showed that free soluble phenolic extracts chelated Fe^2+^ more effectively in a dose-dependent fashion (0–80 µg/mL) than the bound phenolic extracts [56].

In a different study, lipid peroxidation generation was significantly inhibited by the reduction in the level of MDA [18]. A study by Salahudeen and Bolaji [57] revealed that the *n*-hexane fraction of *C. volubile* leaves had an antioxidant effect on DPPH in a dose-dependent manner. Similarly, this finding corroborates the report by Molehin and co-workers [58] on the ameliorative effect of the methanolic extract leaves of *C. volubile* against doxorubicin (DOX)-induced nephrotoxicity in rats. As highlighted by the report of Yang et al. [59], the formation of free radicals, oxidative damage, and lipid peroxidation of the membranes were assumed to be major factors contributing to DOX nephrotoxicity. In this study, the extract was able to boost the antioxidant capability of the depleted endogenous renal antioxidant enzymes (glutathione and catalase) significantly, with a concomitant reduction in the malondialdehyde level. *C. volubile* leaf extracts exerted antioxidant effects by scavenging free radicals, reducing oxidative stress, and restoring the antioxidant defense systems [60]. The results from both the in vitro and in vivo studies highlighted in this section indicate that this plant could be useful in the development of a new antioxidant drug for the management of oxidative stress-induced diseases [58,61].

### 6.3. Antimicrobial Activity

Long before the discovery of humanity, microbes have been in existence [62]. These microbes are important to man but their roles in the pathogenesis of diseases have outshined their merits to man. The prevalence of infectious diseases caused by multi-drug-resistant bacteria has posed a high risk to public health [63]. Over the years, synthetic antibiotics have been utilized for the treatment of bacterial infections, however, the recent occurrence of antibiotic resistance, high-cost implications, and adverse effects limit the use of synthetic antibiotics [64], and hence, these reasons have encouraged the search for cost-effective alternative antimicrobial activity of plant-origin against the resistant bacterial strains [65]. On the other hand, plants have been explored for the treatment of different bacterial infections in most developing countries of the world [66]. The idea is that plants have healing potential and, indeed, scientific validation of these folk claims have increased over time. As a result, several folk herbs used in the treatment of bacterial infections have been well documented in the literature. Among the popular plants used traditionally, *C. volubile* has gained considerable attention. Despite the multipurpose folk uses of the plant, there is still a dearth of information about its antimicrobial activity. In 2016, a study carried out by Jeff-Agboola and Awe investigated the antifungal activity of its solvent (cold-water, hot water, and ethanol) extracts against *Aspergillus flavus* using a well diffusion method and minimum inhibitory assays [67]. In the study, the cold water and ethanolic extracts were recorded to exert fungistatic and fungicidal effects with a zone of inhibition of 13.00 mm and 15.00 mm, respectively. The hot water extract was inactive, and it could be possible that the temperature of the hot water negatively affected the bioactive compounds in the extract. For the minimum inhibitory concentration (MIC) results, the cold-water extract exhibited fungistatic activity against *A. flavus* at 25 mg/mL, while the ethanolic extract showed fungicidal activity at 50 mg/mL. There have been limited reports on the antimicrobial activity of *C. volubile* and, therefore, there is a need to investigate the potential of solvent extracts of this plant against other virulent bacteria and fungi to disentangle its prospects in the treatment of pathogenic diseases [17]. The fact that a plant extract exhibits activity is of interest [62], but it is only a preliminary piece of data; therefore, further studies on the isolation of pure compounds and possible mechanisms of action that would provide a better understanding of the activities of the plant should be encouraged.

### 6.4. Neuroprotective Activity

Neurodegenerative diseases such as Alzheimer’s Disease (AD), Parkinson’s disease (PD), and multiple sclerosis (MS) are caused by a gradual loss in cognitive functions and sensory dysfunction that leads to slow neuronal death [68]. The pathological processes of neurodegenerative diseases include oxidative stress, mitochondrial dysfunction, genetic factors, inflammation, and apoptosis, which result in neuronal degeneration in PD [69]. A study by Olcese et al. [70] highlighted that severe lipid peroxidation and dopaminergic neurons lead to the destruction of cholinergic neurons in AD [71]. An enzymatic antioxidant, such as superoxide dismutase (SOD) [71], and non-enzymatic antioxidant, such as total thiol groups, are present in the brain. The central nervous system (CNS) comprises high polyunsaturated fatty acids that are usually prompt to peroxidation [72]. The relatively low antioxidant enzymes in the brain as compared to other tissues make the former more prone to oxidative damage [73].

In traditional medicine, different parts of plants have been explored in the management of several neurodegenerative diseases [74]. Examples of these plants include *Withania somnifera* [75]. These plants have demonstrated strong acetylcholinesterase inhibitory potential. Furthermore, reduction in pro-inflammatory cytokines (IL-1β, IL-6, TNF-α) and total nitrite generation are some of the main neuroprotective mechanisms of bioactive compounds of plant origin [74]. The neuroprotective potential of *C. volubile* has not been studied extensively, as Oboh and colleagues [42] gave the first report on the neuroprotective potential of *C. volubile*. The study showed that phenolic-rich extracts from *C. volubile* leaves demonstrated inhibitory effects against monoaminergic [monoamine oxidase (MAO)] and cholinergic [acetylcholinesterase (AChE) and butyrylcholinesterase (BChE)] enzymes’ activities and pro-oxidant [Fe^2+^ and quinolinic acid-(QA)]-induced lipid peroxidation in rats’ brain. The study concluded that the inhibitory activities demonstrated by the leaf extracts of *C. volubile* could be the major reason why the plant is used in the folk settings for the management of neurodegenerative and related diseases. Further research should be tailored towards the isolation of the bioactive compounds and their mechanisms of action can provide more data that will be relevant for the development of neuroprotective agents from this plant.

### 6.5. Antiviral Activity

Several viral infections are reported annually worldwide [76]. A study has shown that some standard antiviral drugs, such as ribavirin and interferon, show high antiviral activity in vitro, but they are not effective in vivo. Presently, most of the viral infections have no cure and vaccination is only available for a few viral diseases, and hence, there has been an upsurge in the death rate resulting from the viruses [77]. Besides, the existing antiviral drugs are associated with side effects, and thus, natural products of plant origin have been serving as an alternative in folk settings for the treatment of viral infections and diseases because of their availability, safety, and cost-effectiveness [76]. Therefore, several medicinal plants such as *Capparis spinosa*, *Ficus benjamina*, *Lilium candidum*, *Phyllanthus acidus*, and *Quercus persica* [78,79,80,81] have been assessed for their antiviral activity to validate their folk claims.

As the COVID-19 pandemic spreads globally, research scientists and policymakers are making continual efforts to curtail the transmission by consistent public health interventions, such as through the use of a nose mask, washing of hands, social distancing, testing of suspected cases, and isolation [82,83]. Interestingly, repurposed drugs, such as remdesivir, ritonavir, chloroquine, and lopinavir, among others, are being used in the management of COVID-19 infection [84]. In addition, the use of supplements that can boost the host immune system has been documented as a good strategy to fight the virus [85]. Unfortunately, research on the exploration of medicinal plants for the development of antiviral drugs against coronavirus is slow, as much attention has been currently geared towards vaccine development globally [86,87]. Erukainure and colleagues [86] have investigated the inhibitory potential of harpagide 5-*O*-β-D-glucopyranoside, a new iridoid glycoside isolated from methanolic leaves of *C. volubile* against SARS CoV-2.

### 6.6. Anti-Inflammatory Activity

Inflammation is a physiological response that occurs in the body because of infection, irritation, or injury to safeguard the body and speed up the recuperation development [88]. However, unchecked inflammation can lead to chronic inflammatory diseases. In addition, excessive production of leukotrienes and prostaglandins via lipoxygenase (LOX) and cyclooxygenase (COX) pathways can initiate and maintain injured cells. This process involves the activation of plasma proteins and leukocytes at the site where the infection occurs to fight against the infectious agent [89]. As previously highlighted by the report of Iwalewa et al. [90], most infections or diseases occur via chronic inflammation, where the body cannot repair the damaged cells, and hence, they degenerate. To develop anti-inflammatory drugs, a biocompatibility of the product must be thoroughly ascertained.

Inhibition of xanthine oxidase decreases uric acid production, whose presence confirms gout medical condition [91]. Some medicinal plants have demonstrated the ability to inhibit some enzymes, such as xanthine oxidase, implicated in gout arthritic conditions through the scavenging of ROS, which is generated as a result of inflammatory responses, as well as uric acid deposit in the joints [92]. Anti-inflammatory agents usually carry out their functions by stabilizing the red blood cells induced by hypotonic lysis [88]. A report by Oyedapo et al. [93] stated that some natural compounds of plant origin can reverse and stabilize biological membranes when exposed to stress. For example, different organic solvents (ethyl acetate, methanolic, and petroleum ether) extracts of *C. volubile* demonstrated higher anti-inflammatory effects than the standard anti-inflammatory drug, diclofenac sodium [23]. Findings from this study justified the use of *C. volubile* to suppress pain and other inflammation symptoms in folk medicine. In another study, documented by Amole et al. [12], on the anti-inflammatory effect of hydroethanolic extract of *C. volubile* leaves in rodents, dose-dependent inhibition of edema development in carrageenan-induced inflammation and cotton-pellet-induced granuloma formation in rats was observed. The study also noted that the extract was not acutely toxic orally and this means that the biological effect could be mediated through peripheral and central mechanisms comprising the activation of dopaminergic receptors. Similarly, the report of Olarenwaju et al. [88] described the anti-inflammatory effect of ethanolic leaf extract against carrageenan-induced rat paw edema. The study revealed the inhibition of inflammation biomarkers such as xanthine and lipoxygenase. It is worthy of note that the extract inhibited cyclooxygenase, an enzyme implicated in prostaglandins formation, and xanthine oxidase, while reducing rat paw edema in a dose-dependent manner. Therefore, the above scientific claims validate the exploration of the plant for the folk treatment of several inflammation-related conditions [94].

### 6.7. Hepatoprotective Activity

Bioactive compounds (phenolics, tannins, flavonoids, alkaloids, terpenoids, saponins, and sterols) from plants have been acknowledged to possess several therapeutic potentials, such as hepatoprotective activity via antioxidant capacity [95,96]. From the literature, in vitro and in vivo models have been exploited to investigate the protective role of some crude extracts or isolated compounds against the toxic effect of some toxins on the liver cells [96,97]. For example, the protection against liver damage induced by carbon tetrachloride (CCl_4_) has been extensively studied [98]. The study carried out by Molehin et al. [16] reported the hepatoprotective role of methanolic extract of *C. volubile* leaves against oxidative stress and liver toxicity induced by CCl_4_ in rats. The study showed that *C. volubile* extract exhibited an ameliorative effect on the hepatic damaged caused by the toxin (CCl_4_). This was buttressed by the decrease in the activities of liver biomarker enzymes, including aspartate aminotransferase (AST), alkaline phosphatase (ALP), alanine aminotransferase (ALT), and other biomarkers of hepatic damage [99]. Remarkably, the activities of endogenous liver antioxidant enzymes, such as superoxide dismutase, glutathione (GSH), glutathione peroxidase and catalase, increased significantly in the experimental rats treated with methanolic leaves extract of *C. volubile*. The findings revealed that the extract restrained mitochondrial membrane pore openings in the insulin resistance condition at low concentrations [100].

### 6.8. Antidiabetic Activity

Type 2 diabetes (T2D) is the most prevalent among all types of diabetes, with high global mortality and morbidity rates [31]. The treatment cost of diabetes is on the high side, especially for low-income earners or rural dwellers in developing countries, as well as in areas with a high prevalence of non-communicable diseases in the health system. The huge burden of other diseases on the weak health system in Africa has been reported as accounting for the high mortality of people with diabetes compared to the advanced countries around the world [101]. The preliminary screening of medicinal plants for antidiabetic activity has been conducted with α-amylase and α-glucosidase inhibition assays [102,103]. This assay is significant as it reveals the delay caused by the plant extracts in the digestion of carbohydrates. The high inhibition rate implies the slow release of glucose into the bloodstream [104]. As highlighted by the report of Robertson and Harmon [105], “the role of oxidative stress in the progression and pathogenesis of T2D and its complications has been attributed to the extremely low level of antioxidants in the pancreatic β-cells”. To buttress this fact, findings of Adiels et al. [106] stated that if oxidative stress is left unimpeded, it can cause dysfunction of the pancreatic β-cell, thus instigating chronic hyperglycemia, as well as dyslipidemia, which is usually observed in the non-obese T2D conditions. Besides, increased hyperglycemia triggers unnecessary free radical production, thereby leading to oxidative stress that is responsible for the cause of most diabetic micro-and macro-vascular complications [107,108].

The existing antidiabetic drugs are expensive, and they are associated with side effects, hence, searching for a safe and cost-effective alternative has become essential. Therefore, the interest in medicinal plants for drug development has been increasing over the years. Besides, in African countries, most people rely on folk medicines for the treatment of diseases such as diabetes [109,110]. It has been reported that some bioactive compounds from certain plants possess antidiabetic activity by inhibiting the enzymes involved in the digestion of carbohydrates [111]. Among the plants used traditionally for managing diabetes, *C. volubile* has gained popularity because of its remarkable medicinal uses [56], such that it is popularly referred to as “magic leaf” in the southwestern part of Nigeria [112]. The study by Adefegha and Oboh [18] reported the inhibition of α-amylase, α-glucosidase, and angiotensin-1 in converting enzymes. Similarly, Molehin [16] documented that the leaf extract of *C. volubile* significantly inhibited α-amylase (IC_50_ = 0.40 mg/mL) and α-glucosidase (IC_50_ = 0.68 mg/mL). In addition, another study conducted by Erukainure et al. [113] highlighted the modulatory potential of fatty acids and some chemical fractions from the flower and stem of *C. volubile* against phagocytic oxidative bursts. Besides, the hypoglycaemic activity of protocatechuic acid and ethyl acetate fractions from the leaves was also reported [114]. Other fractions, including methanol, dichloromethane, butanol, and aqueous, have also demonstrated anti-diabetic properties in T2D rats [30]. These findings were corroborated by Molehin et al. [60], who reported the antihyperglycemic effect of aqueous leaves of *C. volubile* in streptozotocin (STZ)-induced diabetic rats. Recently, Molehin and colleagues evaluated the in vivo antidiabetic effect of *C. volubile* leaves in streptozotocin (STZ)-induced diabetic rats. They found that among the several phytochemicals identified in the studied extract, rutin could be the leading bioactive compound responsible for the biological activity of *C. volubile*, thereby supporting the folk use of this plant [58]. The scientific claims justify that polyphenolic compounds can significantly reduce elevated blood glucose by instigating the increase in glucose uptake, as well as glycogen synthesis, thus reducing post-prandial hyperglycemia [18].

### 6.9. Anti-Hypertension Activity

Hypertension is a life-threatening health problem that negatively affects other cardiovascular diseases, and it is a major cause of premature death worldwide [115,116]. It has been documented that free-radical-induced oxidative damage to a blood vessel’s endothelial cells can hamper the elasticity of the vessel, thereby leading to hypertension or other related cardiovascular conditions [117]. Several synthetic drugs are currently being used to manage clinical hypertension; however, their long usage has been associated with some dissatisfaction, hence, a search for alternatives with fewer side effects has become imperative. This has prompted the use of medicinal plants for managing hypertension, which has been on the increase in recent times. Angiotensin-I-converting enzyme (ACE) is the significant enzyme responsible for the control of blood pressure. It converts angiotensin I to angiotensin II, which is a potent vasoconstrictor. The inhibition of ACE activity is a good strategy to lower blood pressure in a hypertensive patient [118,119,120]. The positive correlation between phenolics and ACE inhibition has been documented in the literature. The catalytic site of ACE contains zinc metal, and thus, it is referred to as metalloprotein. The study of Umamaheswari et al. [121] reported that phenolics can chelate the zinc ions at the catalytic site of ACE and form strong hydrogen bonds with the amino acid residues at the active site of the enzyme, thus inhibiting it. Furthermore, the study of Adefegha and Oboh [18] acknowledged the in vitro inhibitory potential of phenolic extracts of *C. volubile*. The study observed that the bound phenolic extract showed significantly (*p* < 0.05) higher ACE inhibition than the free soluble phenolic extract. Overall, this study justified why phenol-rich foods have been recommended for the management of hypertension and other cardiovascular diseases [122]. The antihypertensive effect demonstrated by the plant extracts could be through antioxidant mechanisms that scavenge free radicals and chelate transition metal ions. The plant extracts contain some phytochemical compounds with proven pharmacological potentials, thus making it effective in the management of hypertension. Furthermore, Akinpelu et al. [123] investigated the antihyperlipidemic effect of ethanolic extract of *C. volubile* leaves in Wistar rats. The results revealed some significant phytochemical compounds that could be responsible for the biological effect. It was also observed that the levels of total low-density lipoprotein (LDL), triacylglycerides, and cholesterol were lower in the animal group treated with the studied extract as compared with the standard drug, atorvastatin. The findings justified the reason why *C. volubile* is traditionally referred to as a magic leaf. Thus, the isolation of pure compounds that could be used in the development of new antihypertensive drugs from this plant should be given attention. The summary of pharmacological potentials of *C. volubile* are depicted in Table 1.

### 6.10. Other Relevant Activities

Olorundare et al. [125] reported that ethanolic extracts of *C. volubile* leaves significantly attenuated cardiotoxicity induced by doxorubicin (DOX) in rats. As highlighted by the report of Tacar et al. [126], DOX induced oxidative stress in the kidneys of rats with evidence of an increase in lipid peroxidation and an alteration in the antioxidant status indices. In the study of Olorundare and co-workers [125], they observed that the extract was able to ameliorate the cardiac tissue oxidative stress markers in the studied rats through an antioxidant mechanism by scavenging the free radicals generated by DOX toxicity. Similarly, in another study investigated by Olorundare and colleagues, for the first time, ethanolic extract of *C. volubile* leaves significantly decreased trastuzumab (TZM)-induced cardiotoxicity in rats, and its cardioprotective effect was recorded to be facilitated through its antioxidant ability, which stimulates free radical scavenging and inhibits lipid peroxidation [127]. Also, Adeneye et al. [128] confirmed the protective and therapeutic potentials of ethanolic extract of *C. volubile* leaves in trastuzumab-intoxicated Wistar rats. The findings corroborated the report of Molehin [129], who documented the ameliorative potential of methanolic extracts of *C. volubile* leaves against DOX-induced nephrotoxicity in rats. It was noted that DOX injection triggered a significant increase in serum creatinine and urea levels. Also, the renal antioxidant enzymes decreased considerably, while the increase in malondialdehyde level indicates that the toxicity caused lipid peroxidation. Interestingly, treatment of the rats with the studied extract restored antioxidant status, attenuated oxidative stress, and improved kidney function markers.

## 7. Safety Concerns Regarding the Use of the Plant (Toxicity Studies)

Toxicological study is a significant aspect that cannot be neglected in the formulation and development of new drugs for therapeutic uses [130]. This is because the safety of medicinal herbs must be ascertained; hence, it is necessary to determine the concentration that will be safe for human use [131]. As the use of medicinal plants for the treatment of different human illnesses is gaining attention, their safety has become a major concern for scientists and health professionals because of the mode of preparation of herbal mixtures by traditional healers and lack of proper knowledge of the dosage that will not be toxic to human beings [132]. During growth, plants can produce secondary metabolites (phytochemicals), which can be extracted with different solvents. These phytochemicals possess different pharmacological potentials; however, they could be toxic at a high concentration [132]. The toxicology study of several plants with significant therapeutic potentials has been reported in the literature [133]. Likewise, some researchers have investigated the toxicity of the solvent extracts of *C. volubile* to ascertain its safety. For example, the study of Akinpelu et al. [123] reported on the acute toxicity of the ethanolic extract from the leaves of *C. volubile* in rats. Another study, conducted by Erukainure et al. [27], also documented the cytotoxic effect of the dichloromethane fraction of *C. volubile* flowers and their findings revealed that the extract suppressed the proliferation of the CC-1 cell line in a dose-dependent manner.

## 8. Future Perspectives and Conclusions

Although, *C. volubile* has exhibited remarkable pharmacological, nutritional, and ethnomedicinal potentials, as confirmed by scientific evidence, there is a dearth of information on the diversity of microbes residing in the plant tissues. Endophytes are noted for their ability to produce a wide range of secondary metabolites, characterized by diverse pharmacological potentials [134,135]. Some of the therapeutic potentials of endophytic microbes, as recently articulated in a review by Falade et al. [135], include anticancer, anti-inflammatory, antioxidant, antimalarial, antidiabetic, neuroprotective, and antiviral properties. A notable therapeutic agent from endophytes is taxol, a chemotherapeutic agent for treating cancer, which was isolated from plants of “*Taxus*” or “*Taxodium* spp.” [136,137,138]. Other endophyte-derived compounds with therapeutic attributes have been documented by Falade et al. [135]. Besides, some endophytic microbes are known to produce metabolites “analogous” to their host, while others may produce secondary metabolites different from that of the host plant. Considering the multiple medicinal uses and the documented pharmacological properties of *C. volubile*, it can be hypothesized that endophytes from the plant may be promising sources of bioactive compounds for the development of novel therapeutics. It is worthy of note that there is currently no record of bioactive compounds from endophytes hosted by *C. volubile* in the literature. Therefore, research efforts should be geared towards this direction by exploiting the metagenomics approach to study the microbial community in *C. volubile* to discover novel secondary metabolites of pharmacological significance.

In addition, nanoparticles synthesized from plant extracts have recently gained considerable interest perhaps, due to the enormous medicinal properties of such plants. The exploitation of phytochemicals in the development of nanoparticles has created a synergy between natural products and nanotechnology [139,140]. Also, the development of nanoparticles from plant extracts is eco-friendly [141]. Phytoconstituents have been regarded as efficient reducing agents for the synthesis of nanoparticles [142]. Nanoparticles synthesized from plants offer diverse pharmacological activities, including antimicrobial, antioxidant, anticancer, and antimalarial properties [143]. Besides, nanotechnology plays an important role in delivering drugs to specific targets in the body [141]. Nanoparticles have been synthesized from a wide range of medicinal plants including *Salvia officinalis* [139], *Oedera genistifolia* [141], *Lantana camara* [144], *Zanthoxylum chalybeum* [145], and *Feijoa sellowiana* [146] among others. However, data are scarce on the synthesis of nanoparticles from *C. volubile* or its phytoconstituents. Besides, the enormous medicinal properties of the plant would foster its candidature for the green synthesis of nanoparticles for biomedical applications. Therefore, exploration of *C. volubile* for the development of nanotherapeutics is hereby suggested.

Conclusively, this present review highlighted some reported pharmacological, nutritional, and ethnomedicinal potentials of *C. volubile*. The plant has great potential to be used as a reference source for the isolation of lead compounds that could be used in the development of food supplements that can be used against human diseases. Most of the reported studies on *C. volubile* did not highlight any potential side effects if its consumption is not abused. Therefore, proper awareness needs to be created on the therapeutic significance of this underutilized vegetable so that it can be properly exploited as a source of both micronutrient and macronutrients in tackling some diseases and malnutrition facing some rural dwellers in developing countries around the world. However, extensive in vivo study is highly imperative to elucidate the mechanisms of action of the isolated compounds. Furthermore, pharmacokinetic studies must be conducted to investigate the unexploited potential of the plant.

## Figures and Tables

**Table 1:** Pharmacological potentials of *C. volubile* reported from previous studies in the literature.

Pharmacological Activity	Mechanism of Actions	Reference
Antioxidant	Free radical scavenging and chelation of metal ions involved in redox metabolism; increase in endogenous antioxidant enzymes, such as GSH level, SOD, catalase, and GPx activities with a concomitant reduced.	[16,25,35]
Antidiabetic	Inhibition of α-glucosidase and amylase, key enzymes linked to T2D.	[25]
Anticancer	Inhibits cell proliferation, arrests cell cycle progression, down-regulates MMP-9 expression, and attenuates oxidative stress.	[25]
Antihypolipidemia	Lowers plasma cholesterol, LDL, vLDL, and triglyceride levels, as well as increases HDL level.	[16,123]
Antihypertensive	Inhibits ACE by chelating its hydroxyl groups with the Zn^2+^ moiety of ACE and inhibiting vasoconstriction of the blood vessels.	[25,124]
Hepatoprotective	Decreases liver biomarker enzymes such as AST, ALT, ALP, and TB levels; improves hepatic architecture.	[16,25]
Antiinflammatory	Quenches polymorphonuclear neutrophils’ respiratory oxidative bursts; suppression of T-cell proliferation; inhibition of in vitro lipoxygenase, cyclooxygenase, and xanthine oxidase.	[25,88]
Neutroprotective	Inhibition of cholinergic and monoaminergic enzymes such as acetylcholisterases, modulation of the redox homeostasis, mitigates against oxidative stress.	[25,42]
